# Emotion dysregulation as cross-disorder trait in child psychiatry predicting quality of life and required treatment duration

**DOI:** 10.3389/fpsyt.2023.1101226

**Published:** 2023-07-20

**Authors:** Margreet Bierens, Catharina A. Hartman, Helen Klip, Stijn Deckers, Jan Buitelaar, Nanda Rommelse

**Affiliations:** ^1^Karakter Child and Adolescent Psychiatry, University Center, Nijmegen, Netherlands; ^2^Department of Psychiatry, University of Groningen, Groningen, Netherlands; ^3^Department of Pedagogical Sciences, Radboud University Nijmegen, Nijmegen, Netherlands; ^4^Radboud University Medical Center, Donders Institute for Brain, Cognition and Behavior, Nijmegen, Netherlands; ^5^Department of Psychiatry, Radboud UMC, Nijmegen, Netherlands

**Keywords:** emotion dysregulation, quality of life, ADHD, ODD, depression, anxiety

## Abstract

**Background:**

Emotion dysregulation (ED) is increasingly under investigation as a cross-disorder trait, and is by some considered as the core feature in mental disorders. The aims of this study were to scrutinize the overlapping and distinct characteristics of ED for internalizing, externalizing and neurodevelopmental disorders and to identify the most pertinent ED characteristics to guide clinicians in treatment choice.

**Methods:**

Information on clinical diagnosis (Attention Deficit/Hyperactivity Disorder ADHD, Autism Spectrum Disorder, Oppositional Defiant Disorder/Conduct Disorder, Anxiety and Mood Disorders), ED (measured by the CBCL-Emotion Dysregulation Index), Quality of Life (Qol, measured by the Kidscreen-27), and treatment duration (measured by Electronic Health Records) was retrieved from two large samples of toddlers (1.5–5  year old; *N* = 1,544) and school aged children (6–18 year old; *N* = 7,259). Frequency scores and logistic regression were used to study symptom profiles of ED, as measured with CBCL-EDI, across all disorders. Linear regression was used to determine the predictive value of ED (CBCL-EDI total score) regarding QoL and treatment duration in addition to—and in interaction with—clinical diagnosis.

**Results:**

Across disorders, equal levels of total ED were found, which predicted lower QoL and a longer treatment duration in addition to clinical diagnosis. The majority of items (11/15 and 16/18) were of equal relevance to the disorders; items that were not, largely reflected disorder specific DSM definitions (i.e., externalizing symptoms in ODD/CD and internalizing symptoms in Anxiety and Mood disorders).

**Conclusion:**

ED is a clinically useful cross-disorder trait to predict severity of impairment as well as required treatment duration. In addition, ED is largely composed of shared features across disorders, with certain disorder specific colored elements.

## 1. Introduction

Children and adolescents can be easily overwhelmed by emotions. This holds in particular for children and adolescents with mental disorders. There is increasing research and clinical interest in Emotion Dysregulation (ED) as a cross-disorder trait. ED is commonly defined as emotional impulsivity and deficient emotional self-regulation (1) and in most definitions includes symptoms of heightened reactivity and quickness to angry, anxious or depressed affect (2, 3). Successful emotion regulation is associated with good mental and somatic health outcomes, social relationships, academic performance and work functioning (4, 5). ED may potentially be relevant in understanding the general risk for the development of psychopathology [“p-factor”; (6, 7)] as well as be a relevant target for treatment regardless of disorder (8–10). Indeed, ED is more prevalent in youth with Attention Deficit/Hyperactivity Disorder [ADHD; (2)], Autism Spectrum Disorder [ASD; (11)], Conduct Disorder/Oppositional Defiant Disorder [CD/OCC; (12)], mood disorders (13) and anxiety disorders (14). In addition, ED signals a more persistent treatment course and poorer prognosis (15, 16). Despite the promising transdiagnostic and treatment value of ED, previous findings indicate the complexity of emotion regulation processes and outcomes [e.g., subjective feelings, motivated behavior, physiological reactivity; (17)]. That is, ED unfolds in myriad ways that might have different functional relations with symptom development (18). The strong temporal comorbidity between internalizing and externalizing conditions requires a more nuanced approach to emotion regulation processes in the various developmental stages (17). Therefore, the aims of this study were to scrutinize the overlapping and distinct characteristics of ED for internalizing, externalizing and neurodevelopmental disorders and to identify the most pertinent ED characteristics to guide clinicians in treatment choice. The shared and specific features of parent-reported ED were examined in a large sample of youth (*N* = 8,803; 1.5–18 years) clinically diagnosed with ADHD, ASD, ODD/CD, Anxiety and/or Mood disorders. The predictive relevance of ED was examined in relation to quality of life and treatment duration in addition to—and in interaction with—clinical diagnosis.

## 2. Method

### 2.1. Sample

The sample consisted of *N* = 8,803 children and adolescents who were referred to Karakter Child-and Adolescent Psychiatry in Netherlands, between March 2012 and May 2017. The sample included *N* = 6,299 (71.6%) boys, M_age_ at entry = 9.1 years, SD = 3.7, and *N* = 2,504 (28.4%) girls, M_age_ at entry = 10.7 years, SD = 4.2. Karakter offers academic care and is specialized in the treatment of neurodevelopmental disorders. Clinical DSM-IV-TR (19) and DSM-5 diagnoses [2014 and later; (20)] were established by a multidisciplinary team consisting of a mental health specialist (psychiatrist or clinical psychologist/clinical neuropsychologist) and mental health generalist (nurse practitioner, psychologist). Diagnosis was always based on parent-and teacher-reported information (o.a. prior medical and mental health issues, developmental milestones, school results, behavioral questionnaires) and direct observation of the child in interaction with the mental health specialist. In case diagnostic uncertainty remained, additional information was gathered using direct observations and/or assessments (cognitive evaluation, observation of family interactions, school observation). Only when a consensus diagnosis was reached, the respective DSM-classifications were assigned. This step-wise, consensus diagnosis method is seen as most reliable (21).

The present study was approved by the institutional review board of Karakter Child-and Adolescent Psychiatry on June 20th 2017.

### 2.2. Procedures

All data was derived from ROM and EHR. Cases were pseudo anonymized (e.g., names replaced with a study ID number) and address information was omitted upon withdrawal and therefore no individual health records were assessed and privacy remained secured. Data was directly put into an IBM SPSS statistics 25 file. DSM diagnoses were clustered into 5 main disorder categories: Attention Deficit/Hyperactivity Disorder (ADHD), Autism Spectrum Disorder (ASD), Conduct Disorder/Oppositional Defiant Disorder (CD/ODD), Anxiety disorders {e.g., Anxiety Disorder Not Otherwise Specified (27.2%), Post-Traumatic Stress Disorder (20.5%), Obsessive Compulsive Disorder (1.2%) and Generalized Anxiety Disorder (15.5%), and Mood disorders [e.g., Dysthymia (66.3%) and Mood Disorder Not Otherwise Specified (35.1%)]}.

### 2.3. Measurements

#### 2.3.1. Emotion dysregulation

Emotion dysregulation was operationalized using the 18-item Emotion Dysregulation Index of the Child Behavior Checklist [CBCL-EDI; (22, 23)]. The CBCL-EDI was constructed by 23 clinicians and experts working in the field of psychiatry and ED research, by examining each item of the CBCL and assessed to what degree they found the item relevant for ED. Eighteen items were selected from different subscales (aggressive, social problems, anxious/depressed, withdrawn/depressed and thought problems) and represent both problematic externalizing and internalizing behaviors. Internal consistency was proven to be excellent [Cronbach’s alpha = 0.90; (22)]. In this study, the CBCL *preschool version* (age 1.5–5) and *school age version* (age 6–18) was used. All items were answered on a 3-point Likert scale (i.e., 0 = not true, 1 = somewhat or sometimes true, 2 = very true or often true). Item 18 (*self-harm and suicidal tendencies*), 91 (*contemplating suicide*) and 97 (*threatening people*) are missing from the preschool CBCL-EDI and therefore, the CBCL-EDI for this age group consisted of 15 items instead of 18 (see Table 1). ED data was collected pre-treatment. Internal consistency in the present study was proven to be very good (Cronbach’s alpha =0.82 and 0.85, respectively). See Supplementary Table S1.

**Table 1:** CBCL preschool and school age, age, gender, Kidscreen-27, and treatment minutes by disorder categories.

	ADHD	ASD	ODD/CD	Anxiety
Preschool *N* = 1,544^a^	612 (39.6)	931 (60.3)	133 (8.6)	100 (6.5)
Comorbidity^b^ ADHD	–	158 (17.0)	55 (41.4)	9 (9.0)
ASD	158 (25.8)	–	10 (7.5)	10 (10.0)
ODD/CD	55 (9.0)	10 (1.1)	–	8 (8.0)
Anxiety	9 (1.5)	10 (1.1)	8 (6.0)	–
None	396 (64.7)	753 (80.8)	60 (45.1)	71 (71.0)
Age^c^	4.2 (0.8)	3.5 (1.0)	3.9 (1.1)	4.1 (1.2)
Gender^d^	77.6	79.7	72.9	57.0
CBCL-EDI^e^ (0–30)	11.2 (5.2)	10.8 (5.7)	14.0 (5.7)	12.4 (5.1)
Treatment duration^f^	105.7 (134.5)	94.4 (112.9)	100.3 (134.7)	75.9 (92.9)

	ADHD	ASD	ODD/CD	Anxiety	Mood
School age *N* = 7,259^a^	3,821 (52.6)	3,590 (49.4)	647 (8.9)	1,056 (14.5)	937 (12.9)
Comorbidity^b^ ADHD	–	1,251 (34.8)	355 (54.9)	284 (26.9)	204 (21.8)
ASD	1,251 (32.7)	–	149 (23.0)	269 (25.5)	289 (30.8)
ODD/CD	355 (9.3)	149 (4.2)	–	59 (5.6)	49 (5.2)
Anxiety	284 (7.4)	269 (7.5)	59 (9.1)	–	206 (22.0)
Mood	204 (5.3)	289 (8.1)	49 (7.6)	206 (19.5)	–
None	1727 (45.1)	1,632 (45.4)	35 (5.4)	238 (22.5)	189 (20.1)
Age^c^	9.6 (2.9)	10.4 (3.0)	11.1 (3.3)	11.88 (3.0)	14.0 (2.5)
Gender^d^	76.4	78.1	76.0	49.4	46.0
CBCL-EDI^e^ (0–36)	11.7 (6.3)	13.0 (6.3)	15.5 (6.5)	13.3 (6.0)	13.7 (6.0)
Kidscreen-27^g^ (27–135)	91.5 (0.87)	88.3 (0.87)	89.4 (1.1)	90.0 (0.99)	87.6 (1.0)
Phys wellbeing^g^ (5–23)	17.2 (0.26)	16.2 (0.26)	17.1 (0.34)	16.5 (0.30)	16.3 (0.31)
Psyc wellbeing^g^ (7–35)	22.6 (0.31)	21.5 (0.31)	21.6 (0.39)	21.6 (0.35)	20.6 (0.36)
Aut and Parents^g^ (7–35)	26.5 (0.27)	26.5 (0.27)	25.9 (0.35)	26.7 (0.30)	26.5 (0.31)
Soc sup/Peers^g^ (4–20)	12.8 (0.25)	11.9 (0.25)	12.9 (0.32)	12.9 (0.28)	12.4 (0.29)
School Env^g^ (4–20)	12.2 (0.21)	12.3 (0.21)	11.9 (0.28)	12.3 (0.24)	11.8 (0.25)
Treatment duration^f^	95.8 (123.9)	124.3 (154.5)	132.6 (146.1)	149.2 (174.4)	169.8 (188.0)

a Values represent *N* (%); Numbers do not add up since patients can have more than one diagnostic classification; Mood omitted from preschool sample.

b Values represent *N* (%) of comorbidity of disorder Y in diagnostic group X.

c Values represent mean (SD).

d Values represent % boys.

e Values represent mean (SD).

f Values represent mean hours (SD).

g Values represent estimated marginal means (SE).

#### 2.3.2. Quality of life

Kidscreen-27 was used to assess quality of life (*Physical well-being*, *Psychological well-being*, *Autonomy and Parent relation*, *Peers and social support* and *School environment*) in children and adolescents according to parent reports (24). All items were answered on a 5-point Likert scale (i.e., 1 = not at all/never, 2 = mostly not/almost never, 3 = sometimes/medium, 4 = quite/often, 5 = totally true/always). Low scores on one of the subscales or a low total score indicate low subjective health and well-being. Age norms for Kidscreen-27 range from 8 to 18 years old. Therefore, in the current study, Qol was not assessed for the preschool age group. Qol data was collected pre-treatment.

#### 2.3.3. Treatment duration

Treatment information for all referred children was registered in EHR system *User*. Treatment duration was calculated as the total amount of minutes spend on finalized (in) direct treatments of the child as registered by clinicians. Direct treatment time included diagnostic interviews (cognitive behavioral) therapy sessions, pharmacotherapy, psycho-education and parent counselling. Indirect treatment time included examination of diagnostic interviews or questionnaires, documentation/writing letters and consulting tertiary involved caregivers. As this registration is vital to receive financial compensation for offered services, monthly reminders were sent to clinicians to accurately register their appointments in addition to individual reminders in case certain planned patient appointments had not been registered by the end of the month. Therefore, this information was considered to be complete and accurate. Treatment duration data was collected throughout the treatment course until the EPD-record was closed/the patient was deregistered.

### 2.4. Statistical analyses

IBM SPSS statistics 25 was used for statistical analysis. Dichotomous disorder categories were created for each clinical diagnosis (e.g., ADHD, yes = 1, no = 0). Therefore, children with comorbidities were in more than one disorder category (e.g., ADHD = yes, ASD = yes). Cases were deleted from analysis only when age was entered incorrectly (e.g., parent answered with own age instead of the child’s) or when data was missing for CBCL, Kidscreen-27 or treatment duration (e.g., end of treatment was unknown because of ongoing treatment; Supplementary Tables S2, S3). Analyses were run separately for the preschool age and school age, because the items in the two CBCL versions were not fully compatible. Descriptives were used to provide an overview of demographics, CBCL-EDI, Kidscreen-27 and treatment minutes for the disorder categories. Two separate sets of logistic regression analyses were performed for each of the five dichotomous disorder categories: (1) CBCL-EDI total score as independent variable to compare the relative strength of association between total ED and each disorder category versus all others, (2) each CBCL-EDI item as independent variable to examine the ED aspects that were most distinguishable for each disorder category versus all others. Comparisons between disorder categories were all relative, as participants could fall into more than 1 diagnostic category. For each disorder-category, items were ordered by most prevalent to least prevalent based on frequency scores of “often/clearly present” ratings. In addition, linear regression analyses were performed to predict Qol and treatment duration. First, we evaluated if the CBCL-EDI total score predicted Qol and treatment duration as such. Next, we evaluated if the CBCL-EDI total score had additive predictive value for Qol and treatment duration beyond diagnosis. Finally, we examined if the CBCL-EDI total score predicted Qol and treatment duration more in the context of any of the specific disorder categories by adding the interaction between diagnosis and CBCL-EDI in the regression analyses. Age and gender were entered by default in all regression analyses. Linear regression results were corrected for multiple testing by False Discovery Rate [FDR; (25)].

## 3. Results

### 3.1. Descriptive results of the study sample

In the preschool sample, 39.6% met diagnostic criteria for ADHD, 60.3% for ASD, 8.6% for ODD/CD and 6.5% for Anxiety disorders. Numbers exceed 100% as patients can have more than one diagnosis. Comorbidity-rate was highest for ODD/CD (~55%), followed by ADHD (~35%), Anxiety (~30%) and lowest in ASD (~20%). Age varied over disorder categories groups from 3.5 in ASD to 4.2 in ADHD. In all disorder categories except Anxiety, boys were overrepresented (~75–80%). Treatment duration varied from ~105 h in ADHD to ~75 h in Anxiety. See Table 1.

In the school age sample, ~50% met diagnostic criteria for ADHD, ~50% for ASD, ~10% for ODD/CD, ~15% for Anxiety disorders and ~ 13% for Mood disorders. Comorbidity-rate was highest for ODD/CD (~95%), followed by Anxiety and Mood Disorders (~80%) and lowest in ASD (~55%) and ADHD (~55%). Age varied from ~14 years old in Mood disorders to ~9.5 years in ADHD. In ADHD, ASD and ODD/CD the majority were boys (~77%). In Anxiety and Mood disorders, boys and girls were equally represented. ANCOVA was used to study estimated marginal means for all subscales in disorder categories. Kidscreen-27 subscale levels were equal in all disorder. Estimated marginal means for all subscales were relatively lower in the disorder categories compared to norm scores. See Supplementary Figure S1.

### 3.2. Emotion dysregulation symptom profiles and disorder categories

**Figure 1:**
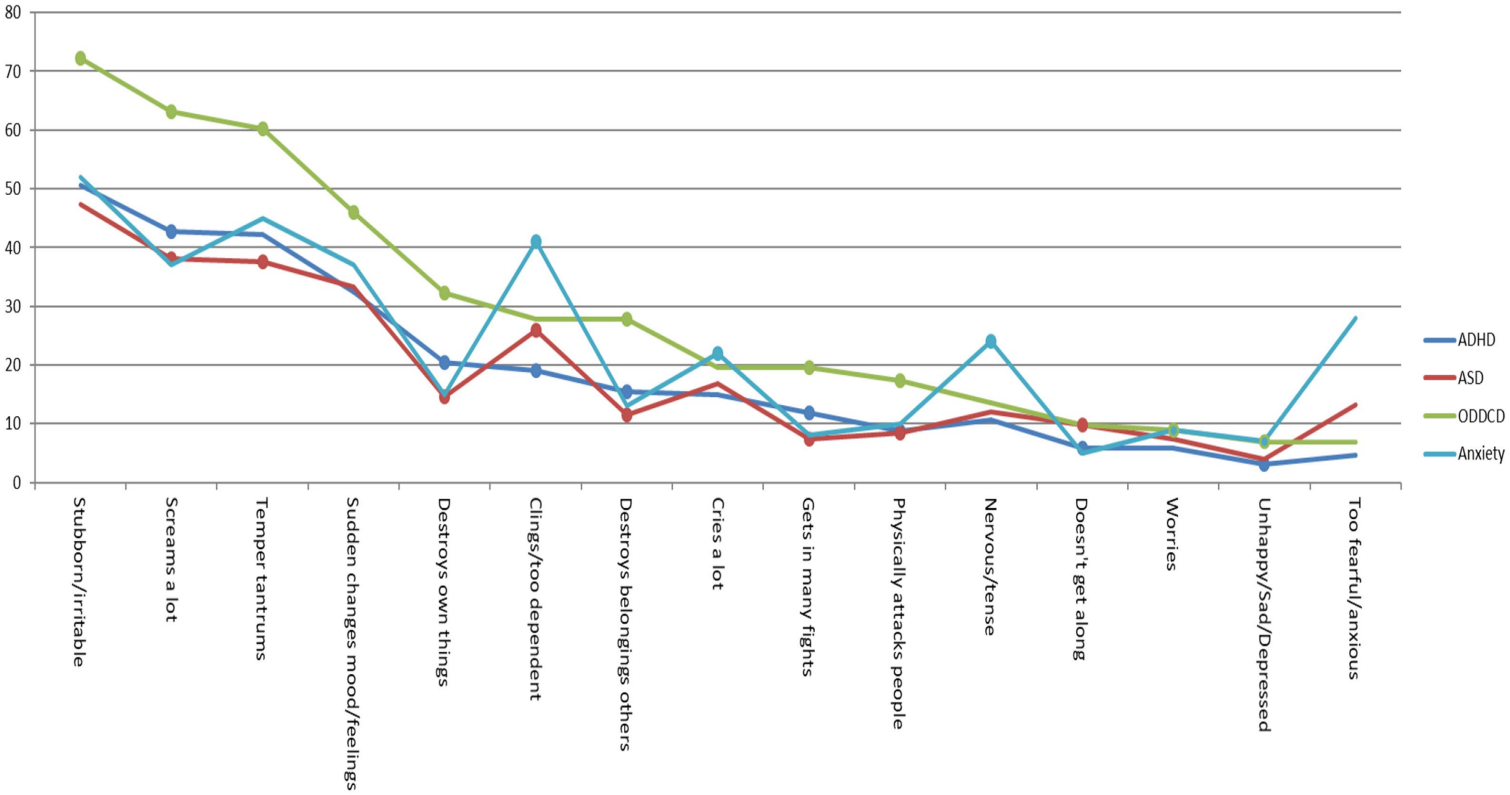
Percentage CBCL-EDI behavior was often/clearly present in preschool age sample. *Dots represent *p* < 0.05 of Exp (B) in univariate regression analysis. See Supplementary Tables S5, S6.

**Figure 2:**
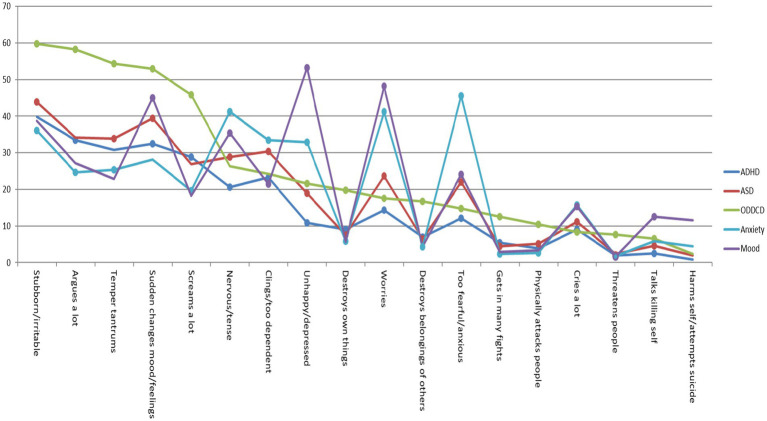
Percentage CBCL-EDI behavior was often/clearly present in school age sample. *Dots represent *p* < 0.05 of Exp (B) in univariate regression analysis. See Supplementary Tables S5, S6.

Logistic regression analysis showed that most CBCL-EDI items were significant in predicting the disorder categories (e.g., ADHD 1 = present, 0 = absent). In the preschool sample, fewer CBCL-EDI behaviors were significant for predicting the disorder category Anxiety (6/15) compared to all other disorder categories (ADHD 10/15, ASD 10/15 and ODD/CD 11/15). “Cries a lot,” “nervous high-strung or tense” and “sudden changes in mood or feelings” were insignificant for predicting 3/4 of the disorder categories. See Figure 1 and Tables 2, 3. In the school age sample fewer CBCL-EDI behaviors were significant for predicting the disorder category Mood (11/18) compared to all others (ADHD 16/18, ASD 15/18, ODD/CD 15/18 and Anxiety 15/18). “Deliberately harms or attempts suicide” was insignificant for predicting 3/5 of the disorder categories. See Figure 2 and Tables 2, 4.

**Table 2:** Logistic regression analyses predicting disorder category by EDI total score.

	ADHD				ASD				ODD/CD				Anxiety			
	Yes = 612		No = 932		Yes = 931		No = 613		Yes = 133		No = 1,411		Yes = 100		No = 1,444	
			95% CI				95%CI				95%CI				95%CI	
	Exp (B)	*p* value	Lower	Upper	Exp (B)	*p* value	Lower	Upper	Exp (B)	*p* value	Lower	Upper	Exp (B)	*p* value	Lower	Upper
CBCL preschool age EDI total^1^	1.0	0.99	0.98	1.0	0.99	0.12	0.97	1.0	**1.1**	**0.001**	1.1	1.1	**1.1**	**0.01**	1.0	1.1
Age	1.1	0.001	1.0	1.1	**0.96**	**0.001**	0.95	0.97	1.0	0.19	0.99	1.0	1.0	0.37	0.99	1.0
Gender	1.1	0.37	0.87	1.5	**1.7**	**0.001**	1.3	2.1	0.83	0.36	0.55	1.2	**0.38**	**0.001**	0.25	0.58

	ADHD				ASD				ODD/CD				Anxiety				Mood			
	Yes = 3,821		No = 3,438		Yes = 3,590		No = 3,669		Yes = 647		No = 6,612		Yes = 1,056		No = 6,203		Yes = 937		No = 6,322	
			95% CI				95%CI				95%CI				95%CI				95%CI	
	Exp (B)	*p* value	Lower	Upper	Exp (B)	*p* value	Lower	Upper	Exp (B)	*p* value	Lower	Upper	Exp (B)	*p* value	Lower	Upper	Exp (B)	*p* value	Lower	Upper
CBCL school age EDI total^1^	**0.96**	**0.001**	0.96	0.97	**1.0**	**0.001**	1.0	1.0	**1.1**	**0.001**	1.1	1.1	**1.0**	**0.001**	1.0	1.0	**1.1**	**0.001**	1.0	1.1
Age	**0.99**	**0.001**	0.99	0.99	1.0	0.69	0.99	1.0	**1.0**	**0.001**	1.0	1.0	**1.0**	**0.001**	1.0	1.0	**1.0**	**0.001**	1.0	1.0
Gender	**1.4**	**0.001**	1.3	1.6	**2.2**	**0.001**	1.9	2.4	**1.6**	**0.001**	1.3	2.0	**0.43**	**0.001**	0.38	0.50	**0.46**	**0.001**	0.40	0.54

1 Child Behavior Checklist preschool age 1.5–5. Classification mood is omitted due to small sample size.

Child Behavior Checklist school age 6–18. Exp (B), odds ratio; predicting disorder category; 95%CI, 95% confidence Interval; ADHD, attention-deficit/hyperactivity disorder; ASD, autism spectrum disorder; ODD/CD, oppositional defiant disorder or conduct disorder; Anxiety, anxiety disorder; Mood, mood disorder.

**Table 3:** Univariate logistic regression analyses predicting disorder category by CBCL1.5–5 EDI items.

	ADHD				ASD				ODD/CD				Anxiety			
	Yes = 612		No = 932		Yes = 931		No = 613		Yes = 133		No = 1,411		Yes = 100		No = 1,444	
CBCL preschool age EDI item^1^			95% CI				95%CI				95%CI				95%CI	
	Exp (B)	*p* value	Lower	Upper	Exp (B)	*p* value	Lower	Upper	Exp (B)	*p* value	Lower	Upper	Exp (B)	*p* value	Lower	Upper
25. Does not get along with other children	**0.74**	**0.001**	0.62	0.88	**1.3**	**0.001**	1.1	1.6	1.2	0.17	0.92	1.6	0.75	0.10	0.53	1.0
10. Clings to adults or too dependent	**0.67**	**0.001**	0.58	0.77	**1.2**	**0.01**	1.0	1.4	1.1	0.34	0.88	1.4	**1.8**	**0.001**	1.3	2.3
13. Cries a lot	0.88	0.08	0.75	1.0	1.0	0.79	0.88	1.1	1.1	0.19	0.92	1.5	**1.4**	**0.02**	1.0	1.9
17. Destroys his/her own things	**1.5**	**0.001**	1.3	1.8	**0.76**	**0.001**	0.66	0.88	**1.6**	**0.001**	1.3	2.1	0.87	0.36	0.66	1.2
18. Destroys things belonging to his/her family/other children	**1.5**	**0.001**	1.3	1.7	**0.71**	**0.001**	0.61	0.83	**2.0**	**0.001**	1.5	2.5	0.87	0.39	0.64	1.1
35. Gets in many fights	**1.5**	**0.001**	1.3	1.8	**0.58**	**0.001**	0.50	0.69	**2.2**	**0.001**	1.7	2.7	0.82	0.26	0.58	1.2
47. Nervous, high strung or tense	0.89	0.13	0.76	1.0	1.0	0.28	0.93	1.2	1.1	0.17	0.93	1.5	**1.7**	**0.001**	1.3	2.3
87. Too fearful or anxious	**0.45**	**0.001**	0.38	0.54	**1.6**	**0.001**	1.3	1.8	**0.72**	**0.02**	0.54	0.97	**2.5**	**0.001**	1.9	3.4
53. Physically attacks people	**1.2**	**0.03**	1.0	1.4	**0.79**	**0.001**	0.67	0.93	**2.1**	**0.001**	1.6	2.6	0.94	0.74	0.68	1.3
66. Screams a lot	**1.3**	**0.001**	1.1	1.5	**0.78**	**0.001**	0.68	0.90	**2.1**	**0.001**	1.6	2.8	0.89	0.40	0.68	1.1
81. Stubborn, sullen or irritable	1.1	0.18	0.95	1.3	**0.83**	**0.02**	0.71	0.97	**2.3**	**0.001**	1.7	3.3	1.1	0.53	0.81	1.5
82. Sudden changes in mood or feelings	1.0	0.72	0.89	1.1	0.97	0.64	0.84	1.4	**1.6**	**0.001**	1.3	2.0	1.1	0.34	0.87	1.5
85. Temper tantrums or hot temper	**1.1**	**0.05**	1.0	1.3	**0.80**	**0.001**	0.69	0.92	**2.1**	**0.001**	1.5	2.7	1.1	0.28	0.89	1.5
90. Unhappy, sad or depressed	**0.61**	**0.001**	0.49	0.76	1.1	0.10	0.97	1.4	**1.3**	**0.04**	1.0	1.8	**1.6**	**0.001**	1.1	2.2
99. Worries	0.81	0.02	0.68	0.97	1.0	0.33	0.92	1.3	**1.1**	**0.001**	0.91	1.5	**1.5**	**0.001**	1.1	2.0

1 Child Behavior Checklist preschool age 1.5–5.

Classification mood is omitted due to small sample size. Exp (B), odds ratio; predicting disorder category; 95%CI, 95% confidence Interval; ADHD, attention-deficit/hyperactivity disorder; ASD, autism spectrum disorder; ODD/CD, oppositional defiant disorder or conduct disorder; Anxiety, anxiety disorder.

**Table 4:** Univariable logistic regression analyses predicting disorder category by CBCL6-18 EDI items.

	ADHD				ASD				ODD/CD				Anxiety				Mood			
	yes = 3,821		No =3,438		yes = 3,590		No = 3,669		yes = 647		No = 6,612		yes = 1,056		No = 6,203		yes = 937		No = 6,322	
CBCL school age EDI item^1^			95% CI				95%CI				95%CI				95%CI				95%CI	
	Exp (B)	*p* value	Lower	Upper	Exp (B)	*p* value	Lower	Upper	Exp (B)	*p* value	Lower	Upper	Exp (B)	*p* value	Lower	Upper	Exp (B)	*p* value	Lower	Upper
3. Argues a lot	**1.1**	**0.001**	1.1	1.2	1.1	0.08	0.99	1.1	**2.7**	**0.001**	2.4	3.1	**0.73**	**0.001**	0.66	0.80	0.92	0.11	0.83	1.0
11. Clings to adults or too dependent	**0.78**	**0.001**	0.73	0.83	**1.6**	**0.001**	1.5	1.7	0.90	0.07	0.81	1.0	**1.4**	**0.001**	1.4	1.6	**0.87**	**0.001**	0.79	0.95
14. Cries a lot	**0.75**	**0.001**	0.70	0.81	**1.2**	**0.001**	1.1	1.3	**0.84**	**0.001**	0.74	0.96	**1.5**	**0.001**	1.4	1.7	**1.7**	**0.001**	1.5	1.9
18. Deliberately harms self or attempts suicide	**0.60**	**0.001**	0.52	0.68	0.97	0.62	0.86	1.0	1.1	0.29	0.91	1.3	1.0	0.76	0.89	1.1	**2.2**	**0.001**	1.9	2.6
20. Destroys his/her own things	**1.1**	**0.001**	1.1	1.3	**0.91**	**0.01**	0.85	0.98	**2.2**	**0.001**	1.9	2.4	**0.86**	**0.01**	0.77	0.97	1.0	0.84	0.89	1.1
21. Destroys things belonging to his/her family/other children	**1.1**	**0.04**	1.0	1.2	**1.0**	**0.05**	0.99	1.2	**2.3**	**0.001**	2.0	2.6	**0.79**	**0.001**	0.70	0.90	0.90	0.17	0.79	1.0
37. Gets in many fights	**1.3**	**0.001**	1.1	1.4	**0.81**	**0.001**	0.74	0.88	**2.7**	**0.001**	2.4	3.1	**0.71**	**0.001**	0.61	0.83	1.0	0.96	0.86	1.1
45. Nervous, high strung or tense	**0.73**	**0.001**	0.68	0.78	**1.3**	**0.001**	1.3	1.4	0.97	0.56	0.86	0.10	**1.7**	**0.001**	1.5	1.9	**1.3**	**0.001**	1.1	1.4
50. Too fearful or anxious	**0.56**	**0.001**	0.52	0.59	**1.4**	**0.001**	1.3	1.4	**0.81**	**0.001**	0.72	0.91	**2.7**	**0.001**	2.5	3.0	**1.1**	**0.001**	1.0	1.2
57. Physically attacks people	**0.80**	**0.001**	0.74	0.88	**1.3**	**0.001**	1.1	1.4	**2.4**	**0.001**	2.0	2.6	**0.78**	**0.001**	0.67	0.90	0.97	0.71	0.83	1.1
68. Screams a lot	**1.1**	**0.001**	1.0	1.2	1.0	0.10	0.99	1.1	**2.1**	**0.001**	1.9	2.4	**0.82**	**0.001**	0.75	0.90	0.91	0.06	0.82	1.0
86. Stubborn, sullen or irritable	0.93	0.06	0.87	1.0	**1.3**	**0.001**	1.0	1.4	**2.0**	**0.001**	1.8	2.4	**0.87**	**0.001**	0.79	0.96	1.0	0.42	0.94	1.1
87. Sudden changes in mood or feelings	**0.76**	**0.001**	0.71	0.80	**1.4**	**0.001**	1.3	1.5	**1.7**	**0.001**	1.5	1.9	1.0	0.90	0.92	1.1	**1.3**	**0.001**	1.2	1.5
91. Talks about killing self	**0.64**	**0.001**	0.58	0.71	**1.1**	**0.001**	1.0	1.2	**1.5**	**0.001**	1.3	1.7	1.1	0.09	0.98	1.2	**2.3**	**0.001**	2.1	2.7
95. Temper tantrums or hot temper	0.94	0.07	0.89	1.0	**1.2**	**0.001**	1.1	1.3	**2.2**	**0.001**	1.9	2.5	**0.89**	**0.001**	0.81	0.97	0.93	0.16	0.85	1.0
97. Threatens people	**0.83**	**0.001**	0.74	0.92	**1.1**	**0.001**	1.0	1.3	**3.1**	**0.001**	2.8	3.6	**0.72**	**0.001**	0.59	0.86	**0.90**	**0.30**	0.75	1.0
103. Unhappy, sad or depressed	**0.57**	**0.001**	0.54	0.62	**1.3**	**0.001**	1.2	1.4	**1.2**	**0.001**	1.0	1.3	**1.4**	**0.001**	1.3	1.5	**3.0**	**0.001**	2.6	3.3
112. Worries	**0.61**	**0.001**	0.57	0.66	**1.2**	**0.001**	1.1	1.3	**0.82**	**0.001**	0.73	0.91	**1.8**	**0.001**	1.6	1.9	**2.2**	**0.001**	2.0	2.5

1 Child Behavior Checklist school age 6–18.

Exp (B), odds ratio; predicting disorder category; 95%CI, 95% confidence Interval; ADHD, attention-deficit/hyperactivity disorder; ASD, autism spectrum disorder; ODD/CD, oppositional defiant disorder or conduct disorder; Anxiety, anxiety disorder; Mood, mood disorder.

Frequency scores (e.g., 2 = often/clearly) showed that in the preschool sample, CBCL-EDI behaviors “stubborn/irritable,” “screams a lot,” “temper tantrums” and “sudden changes in mood/feelings” were the most frequent in all disorder categories. “Clings/too dependent” and “Nervous/tense” were relatively more frequent in disorder category Anxiety compared to all others. See Figure 1 and Table 5. In the school age sample, “stubborn/Irritable,” “sudden changes in mood/feelings,” “temper tantrums” and “argues a lot” were the most frequent in all disorder categories. “too fearful/anxious” was relatively more frequent in disorder category anxiety and “nervous/tense,” “worries” and “unhappy/depressed” were relatively more frequent in both Anxiety and Mood. Externalizing behaviors “destroys own things,” “destroys belongings of others” and “gets in many fights” were more frequent in ODD/CD compared to all other disorder categories. See Figure 2 and Table 5. In both samples, the most frequent CBCL-EDI behaviors were mainly similar, with some disorder specific exceptions (e.g., “too fearful/anxious” in anxiety, “unhappy/depressed” in mood).

**Table 5:** Frequency/percentage CBCL-EDI is often/clearly present in preschool and school age group.

No.	Description	ADHD	ASD	ODD/CD	Anxiety
		*N* = 612 (%)	*N* = 931 (%)	*N* = 133 (%)	*N* = 100 (%)
3	Argues a lot	36 (5.9)	91 (9.8)	13 (9.8)	5 (5.0)
11	Clings to adults or too dependent	117 (19.1)	241 (25.9)	37 (27.8)	41 (41.0)
14	Cries a lot	91 (14.9)	156 (16.8)	26 (19.5)	22 (22.0)
18	Deliberately harms self of attempts suicide	–	–	–	–
20	Destroys his/her own things	125 (20.4)	136 (14.6)	43 (32.3)	15 (15.0)
21	Destroys things belonging to his/her family or others	95 (15.5)	107 (11.5)	37 (27.8)	13 (13.0)
37	Gets in many fights	72 (11.8)	68 (7.3)	26 (19.5)	8 (8.0)
45	Nervous, high-strung, or tense	65 (10.6)	112 (12.0)	18 (13.5)	24 (24.0)
50	Too fearful or anxious	29 (4.7)	123 (13.2)	9 (6.8)	28 (28.0)
57	Physically attacks people	53 (8.7)	78 (8.4)	23 (17.3)	10 (10.0)
68	Screams a lot	262 (42.8)	355 (38.1)	84 (63.2)	37 (37.0)
86	Stubborn, sullen, or irritable	306 (50.6)	441 (47.4)	96 (72.2)	52 (52.0)
87	Sudden changes in mood or feelings	199 (32.5)	310 (33.3)	61 (45.9)	37 (37.0)
91	Talks about killing self	–	–	–	–
95	Temper tantrums or hot temper	258 (42.2)	349 (37.5)	80 (60.2)	45 (45.0)
97	Threatens people	–	–	–	–
103	Unhappy, sad, or depressed	19 (3.1)	36 (3.9)	9 (6.8)	7 (7.0)
112	Worries	36 (5.9)	68 (7.3)	12 (9.0)	9 (9.0)

No.	Description	ADHD	ASD	ODD/CD	Anxiety	Mood
		*N* = 3,821 (%)	*N* = 3,590 (%)	*N* = 647 (%)	*N* = 1,056 (%)	*N* = 937 (%)
3	Argues a lot	1,279 (33.5)	1,224 (34.1)	377 (58.3)	261 (24.7)	255 (27.2)
11	Clings to adults or too dependent	886 (23.2)	1,091 (30.4)	157 (24.3)	353 (33.4)	201 (21.5)
14	Cries a lot	343 (9.0)	399 (11.1)	54 (8.3)	167 (15.8)	143 (15.3)
18	Deliberately harms self of attempts suicide	31 (0.8)	67 (1.9)	15 (2.3)	46 (4.4)	109 (11.6)
20	Destroys his/her own things	342 (9.0)	268 (7.5)	128 (19.8)	62 (5.9)	53 (5.7)
21	Destroys things belonging to his/her family or others	268 (7.0)	228 (6.4)	108 (16.7)	45 (4.3)	40 (4.3)
37	Gets in many fights	208 (5.4)	157 (4.4)	81 (12.5)	24 (2.3)	27 (2.9)
45	Nervous, high-strung, or tense	788 (20.6)	1,035 (28.8)	171 (26.4)	436 (41.3)	332 (35.4)
50	Too fearful or anxious	462 (12.1)	789 (22.0)	95 (14.7)	480 (45.5)	226 (24.1)
57	Physically attacks people	148 (3.9)	188 (5.2)	68 (10.5)	27 (2.6)	31 (3.3)
68	Screams a lot	1,104 (28.9)	966 (26.9)	297 (45.9)	207 (19.6)	171 (18.2)
86	Stubborn, sullen, or irritable	1,520 (39.8)	1,576 (43.9)	387 (59.8)	381 (36.1)	364 (38.8)
87	Sudden changes in mood or feelings	1,240 (32.5)	1,416 (39.4)	342 (52.9)	403 (38.2)	422 (45.0)
91	Talks about killing self	97 (2.5)	165 (4.6)	42 (6.5)	62 (5.9)	118 (12.6)
95	Temper tantrums or hot temper	1,177 (30.8)	1,214 (33.8)	351 (54.3)	267 (25.3)	215 (22.9)
97	Threatens people	77 (2.0)	85 (2.4)	49 (7.6)	19 (1.8)	15 (1.6)
103	Unhappy, sad, or depressed	415 (10.9)	682 (19.0)	140 (21.6)	347 (32.9)	499 (53.3)
112	Worries	549 (14.4)	852 (23.7)	114 (17.6)	436 (41.3)	452 (48.2)

### 3.3. Emotion dysregulation and quality of life

#### 3.3.1. Linear regression predicting quality of life from emotion dysregulation

Linear regression was used to predict Qol from ED. First, ED was used to predict overall QoL (Kidscreen-27 total score). More severe ED predicted lower Qol (β = −0.49, *p* = 0.001). Adding disorder category and the interaction between disorder category and ED, revealed no significant interaction effects, indicating the predictive effect of ED regarding Qol was similar across disorders. Adding disorder category to predict Qol in specific domains (five Kidscreen-27 subscales), was followed by the examination of the interaction between ED and disorder category in predicting Qol in each domain. After correction for multiple testing by FDR, the predictive effect of ED was mostly similar across disorders regarding Qol in different domains. A small number of interactions remained significant for predicting a Qol domain. A higher level of ED predicted poorer Qol in Social support/peers in children with ADHD (β = −0.20, q < 0.01) and ASD (β = −0.13, q < 0.01), but not in ODD/CD, anxiety and mood (β = −0.01; 0.01; 0.20, *p* > 0.1). A higher level of ED predicted poorer Qol in School environment in children with ADHD (β = −0.13, q < 0.01) and anxiety (β = −0.27, q < 0.01) but not in ASD, ODD/CD, and mood (β = −0.02; 0.01; 0.01, p > 0.1). See Table 6.

**Table 6:** Linear regression analyses predicting Kidscreen total scores and subscales by CBCL-EDI, age, gender, and disorder category.

	ADHD		ASD		ODD/CD		Anxiety		Mood	
	*β*	*p* value	*β*	*p* value	*β*	*p* value	*β*	*p* value	*β*	*p* value
Kidscreen total score										
CBCL-EDI	**−0.48**	**0.001**	**−0.50**	**0.001**	**−0.51**	**0.001**	**−0.49**	**0.001**	**−0.49**	**0.001**
Disorder category	**0.09**	**0.001**	**−0.10**	**0.001**	0.01	0.18	0.02	0.12	**−0.11**	**0.001**
ED * diagnosis	−0.01	0.40	0.02	0.09	0.02	0.06	−0.01	0.21	0.01	0.24
Age	**−0.30**	**0.001**	**−0.32**	**0.001**	**−0.32**	**0.001**	**−0.32**	**0.001**	**−0.29**	**0.001**
Gender	**0.05**	**0.001**	**0.07**	**0.001**	**0.05**	**0.001**	**0.06**	**0.001**	**0.04**	**0.001**

	ADHD			ASD			ODD/CD			Anxiety			Mood	
	*β*	*p* value	q-value	*β*	*p* value	q-value	*β*	*p* value	q-value	*β*	*p* value	q-value	*β*	*p* value
Physical wellbeing														
CBCL-EDI	**−0.21**	**0.001**		**−0.21**	**0.001**		**−0.23**	**0.001**		**−0.21**	**0.001**		**−0.21**	**0.001**
Disorder category	**0.15**	**0.001**		**−0.13**	**0.001**		**0.06**	**0.001**		**−0.02**	**0.04**		**−0.10**	**0.001**
ED * diagnosis	0.02	0.19		0.01	0.46		0.02	0.13		**−0.02**	**0.04**		0.02	0.19
Age	**−0.31**	**0.001**		**−0.35**	**0.001**		**−0.35**	**0.001**		**−0.34**	**0.001**		**−0.32**	**0.001**
Gender	**0.11**	**0.001**		**0.14**	**0.001**		**0.11**	**0.001**		**0.11**	**0.001**		**0.11**	**0.001**
Psychological wellbeing														
CBCL-EDI	**−0.54**	**0.001**		**−0.58**	**0.001**		**−0.58**	**0.001**		**−0.57**	**0.001**		**−0.56**	**0.001**
Disorder category	**0.12**	**0.001**		**−0.05**	**0.001**		**0.04**	**0.001**		**−0.03**	**0.001**		**−0.16**	**0.001**
ED * diagnosis	−0.02	0.13		0.02	0.07		0.01	0.26		0.001	0.99		0.01	0.20
Age	**−0.27**	**0.001**		**−0.29**	**0.001**		**−0.30**	**0.001**		**−0.29**	**0.001**		**−0.24**	**0.001**
Gender	**0.08**	**0.001**		**0.10**	**0.001**		**0.09**	**0.001**		**0.08**	**0.001**		**0.07**	**0.001**
Autonomy and parents														
CBCL-EDI	**−0.29**	**0.001**		**−0.33**	**0.001**		**−0.30**	**0.001**		**−0.32**	**0.001**		**−0.31**	**0.001**
Disorder category	**−0.04**	**0.001**		0.01	0.54		**−0.05**	**0.001**		**0.07**	**0.001**		0.001	0.88
ED * diagnosis	−0.03	0.06		0.02	0.19		0.01	0.51		0.01	0.60		0.01	0.36
Age	**−0.04**	**0.001**		**−0.03**	**0.03**		**−0.02**	**0.04**		**−0.04**	**0.001**		**−0.03**	**0.03**
Gender	0.01	0.48		0.001	0.79		0.01	0.53		0.01	0.22		0.01	0.67
Social support/peers														
CBCL-EDI	**−0.21**	**0.001**		**−0.28**	**0.001**		**−0.28**	**0.001**		**−0.27**	**0.001**		**−0.27**	**0.001**
Disorder category	**0.12**	**0.001**		**−0.21**	**0.001**		**0.04**	**0.001**		0.02	0.05		−0.01	0.32
ED * diagnosis	**−0.10**	**0.001**	**0.01**	**0.04**	**0.001**	**0.02**	0.01	0.28		0.01	0.41		0.01	0.65
Age	**−0.13**	**0.001**		**−0.15**	**0.001**		**−0.16**	**0.001**		**−0.16**	**0.001**		**−0.15**	**0.001**
Gender	**−0.04**	**0.001**		0.001	0.92		**−0.04**	**0.001**		**−0.03**	**0.01**		**−0.04**	**0.001**
School environment														
CBCL-EDI	**−0.34**	**0.001**		**−0.28**	**0.001**		**−0.29**	**0.001**		**−0.28**	**0.001**		**−0.29**	**0.001**
Disorder category	**−0.06**	**0.001**		**0.05**	**0.001**		**−0.05**	**0.001**		**0.04**	**0.001**		**−0.08**	**0.001**
ED * diagnosis	**0.05**	**0.001**	**0.02**	−0.03	0.09		0.02	0.11		**−0.04**	**0.001**	**0.01**	−0.01	0.65
Age	**−0.27**	**0.001**		**−0.25**	**0.001**		**−0.25**	**0.001**		**−0.26**	**0.001**		**−0.23**	**0.001**
Gender	**−0.02**	**0.04**		**−0.04**	**0.001**		**−0.03**	**0.03**		**−0.02**	**0.04**		**−0.04**	**0.001**

Kidscreen-27 total score; Kidscreen-27 subscales. Child Behavior Checklist school age 6–18; β, standardized regression coefficient; q-value, FDR corrected *p* value. ADHD, attention-deficit/hyperactivity disorder; ASD, autism spectrum disorder; ODD/CD, oppositional defiant disorder or conduct disorder; Anxiety, anxiety disorder; Mood, mood disorder.

### 3.4. Emotion dysregulation and treatment duration

#### 3.4.1. Linear regression predicting treatment duration from emotion dysregulation

Linear regression was used to predict treatment duration from ED. More ED predicted longer treatment duration for preschoolers and school age children (preschool β = 0.19, *p* < 0.001 and school age β = 0.31, *p* < 0.001). Adding disorder category showed that the predictive effect of ED was similar across disorders regarding treatment duration. The interaction between disorder category and ED, revealed few significant interaction effects. After correction for multiple testing, a higher level of ED predicted a longer treatment duration in children with ASD (β = 0.31, q < 0.01) and mood (β = 0.17, q < 0.01), more so than for children with ADHD, ODD/CD, and anxiety (β = 0.04; 0.03; 0.06, q < 0.01). See Table 7.

**Table 7:** Linear regression analyses predicting treatment minutes by CBCL-EDI, age, gender and disorder category in preschool and school age children.

	ADHD		ASD		ODD/CD		Anxiety	
	*β*	*p* value	*β*	*p* value	*β*	*p* value	*β*	*p* value
CBCL-EDI	**0.18**	**0.001**	**0.20**	**0.001**	**0.19**	**0.001**	**0.21**	**0.001**
Disorder category	0.06	0.04	0.03	0.30	−0.05	0.15	−0.05	0.09
ED * diagnosis	0.03	0.32	0.01	0.89	0.05	0.11	−0.02	0.43
Age	0.04	0.13	**0.06**	**0.03**	**0.06**	**0.04**	**0.06**	**0.04**
Gender	0.01	0.04	0.01	0.72	0.01	0.73	0.01	0.85

	ADHD			ASD			ODD/CD			Anxiety			Mood	
	*β*	*p* value	q-value	*β*	*p* value	q-value	*β*	*p* value	q-value	*β*	*p* value	q-value	*β*	*p* value
CBCL-EDI	**0.30**	**0.001**		**0.27**	**0.001**		**0.31**	**0.001**		**0.30**	**0.001**		**0.31**	**0.001**
Disorder category	**−0.07**	**0.001**		**0.06**	**0.001**		−0.01	0.70		**0.07**	**0.001**		**0.12**	**0.001**
ED * diagnosis	−0.01	0.59		**0.04**	**0.001**	**0.02**	−0.01	0.92		0.001	0.82		**−0.05**	**0.001**
Age	**0.03**	**0.04**		**0.04**	**0.001**		**0.04**	**0.001**		**0.03**	**0.01**		0.01	0.55
Gender	**−0.05**	**0.001**		**−0.06**	**0.001**		**−0.05**	**0.001**		**−0.04**	**0.001**		**−0.04**	**0.001**

Treatment minutes – EHR registrated treatment time. Child Behavior Checklist preschool age 1.5–5. Classification mood is omitted due to small sample size; Child Behavior Checklist school age 6–18. β, standardized regression coefficient; *q-value =* FDR corrected *p* value. ADHD, attention-deficit/hyperactivity disorder; ASD, autism spectrum disorder; ODD/CD, oppositional defiant disorder or conduct disorder; Anxiety, anxiety disorder; Mood, mood disorder.

## 4. Discussion

The aims of this study were to scrutinize the overlapping and distinct characteristics of ED for internalizing, externalizing and neurodevelopmental disorders in a large sample of clinically referred youth (*N* = 8,803, 1.5–18 years) and to identify the most pertinent ED characteristics to guide clinicians in treatment choice. The total level of ED-behaviors was comparable across disorders and in both age groups, with several behaviors being highly prevalent across all disorders. At preschool age, ED typically manifested as externalizing behaviors: “stubborn/irritable,” “screams a lot,” “temper tantrums” and “sudden changes in mood/feelings.” Over 30% of pre-schoolers often/clearly showing these behaviors regardless of diagnosis, with highest prevalence rates in preschoolers with ODD. Other ED behaviors were considerably less prevalent and/or more disorder specific, such as “clings/too dependent” and “nervous/tense” (anxiety) or “destroys own things” and “destroys belongings of others” (ODD). At school age, less cross-disorder ED-behaviors were found. Only “stubborn/irritable” was present often/clearly in over 30% of the clinical sample regardless of diagnosis. Other-predominantly externalizing-typical manifestations of ED were “argues a lot,” “temper tantrums,” “sudden changes in mood/feelings” and “screams a lot,” although these behaviors were prototypical and highly prevalent in children/adolescents with ODD/CD (>50%), ASD and ADHD (both >30%) but not for children/adolescents with anxiety or mood disorder. Anxiety and mood disorder were best characterized by internalizing manifestation of ED: “unhappy/sad/depressed,” “worries,” “nervous/tense” and “too fearful/anxious” were present in >30%. Examining specific domains of Qol showed that more ED on top of ADHD or ASD was related to lower Qol in Social support and more ED on top ADHD and anxiety related to lower Qol in the domain School environment. In addition, a longer treatment duration was found for children that experienced more ED on top of ASD and mood disorders. Our findings suggest that ED is an important cross-disorder marker as it is associated with several childhood disorders, characterized by several commonly shared features, present from early age onwards and predicting a lower quality of life and longer treatment duration. These findings are in line with claims for ED as the core feature in childhood onset disorders (26, 27) and its importance in treatment for improving quality of life (10) and overall outcome (16). Moreover, our findings suggest that the existence of externalizing behaviors of ED in early childhood are to be taken seriously by mental health professionals and to be reluctant of calling such behavior “typical temper tantrums.” Although the behaviors in itself are normative, a high frequency based on parental reports should raise clinical concern given the likelihood a non-normative development is ongoing. Moreover, the current study showed that with increasing age, a more internalizing manifestation of ED is unfolding. This may be caused by the fact that the externalizing manifestation of ED in preschoolers elicit more negative, unresponsive or punitive reactions from caregivers (28). This may pave the way for the development of lower self-esteem and feelings of guilt and shame, until finally by late adolescence these become “incorporated” into a stable negative sense of self-worth (29). Developing effective emotion regulation is highly dependent on safe and supportive parent–child relationships (i.e., secure attachment) in which parents provide a safe context and support and stimulate children’s emotion regulation abilities (28). Offering easy-access parent–child interaction interventions to reduce ED at preschool age may be considered an effective strategy to prevent this vicious cycle (30, 31). Furthermore, our findings suggest that ED on top of ADHD and ASD results in lower Qol in social support/peers and not in ODD/CD, anxiety and mood. The fact that ODD/CD, anxiety and mood have emotional symptoms incorporated in its diagnostic criteria, whereas for ADHD and ASD this is not the case (20), likely contributes to this finding. Additionally, we found thawt ED on top of ADHD or anxiety results in lower Qol in school environment. For ADHD, the defining features already put a strain on the teacher-student relationship and the addition of ED behaviors can simply be “too much to handle” for a teacher, resulting in a cumulation of negative school-related experiences and explaining a decreased the likelihood of graduating from high school or college (32). For anxiety, symptoms are predominantly internalizing. Accompanied by externalizing behaviors of ED (e.g., “Stubborn/irritable”), a similar negative teacher-student interaction pattern can develop, forming a risk factor in school disengagement in childhood and adolescence (33). These findings suggest that despite the predominantly disorder specific colorings of ED in school age, ED manifestations that are not “disorder-defining,” deserve clinical concern and intervention to prevent additional significant problems at school that may have a long-lasting impact on life. The main strengths of this study were two large clinical samples that were available for analyses of both pre-schoolers and children and adolescents. All data were derived from a Routine Outcome Monitoring (ROM) system and electronic health records (EHR), and were therefore highly clinically relevant for studying ED. ED was studied in multiple disorders, shedding light on the cross-disorder nature of this concept. Longitudinal data on treatment duration was available to study the predictive value of ED in clinical practice. A limitation was that our study had no typical developing control group. As a result, relative comparisons were made between disorder categories. However, relative comparisons are well suited to compare shared and specific features of ED, which was our aim. Furthermore, the samples originated from a child psychiatry organization specialized in the treatment of children with neurodevelopmental disorders. As a result, youth with ADHD and/or ASD were overrepresented and comorbidity rates were high. However, sensitivity analyses showed that the results were highly similar in a single-disorder subsample (e.g., ADHD only, ASD only) compared to the full sample (e.g., participant could be in two or more diagnostic category), suggesting findings were not substantially influenced by sample characteristics. Another limitation was that clinical diagnoses were not confirmed using structured interviews. However, similarly for all disorders, a golden standard clinical consensus diagnosis was obtained (21). Therefore, it is unlikely that diagnostic method has influenced the main results. When interpreting the Qol results, caution is necessary as this measure was administered to parents of children 8 years and older. Additionally, more detailed information regarding the types of treatments and services-as well as more refined analyses regarding the association between ED and type of treatment-would have been of additive value. However, this information could not accurately be extracted from the EPD due to several reasons (i.e., change in EPD system during the period of studied records, variation in registration procedures across locations within the institute). Also the definition of “direct” and “indirect” treatment underwent changes during the period of studied records. Therefore, we used the total amount of time as most robust indicator of treatment “complexity.” Taken together, this study showed that at preschool age, ED is commonly present in children with non-normal developmental patterns and mostly manifests as cross-disorder externalizing behaviors (e.g., stubborn/irritable, screams a lot, temper tantrums). At school age, a more disorder-specific manifestation of ED emerged, with externalizing behaviors predominating in youngsters with ODD/CD, ADHD and ASD and internalizing in youngsters with anxiety and mood. However, “stubborn/irritable” was one of few ED behaviors present regardless of diagnosis, indexing the inability to flexible adapt to/cope with situations that evoke difficult emotions (e.g., disappointment, humiliation, shame, anxiety). Higher levels of ED related to a lower quality of life and longer treatment duration and disproportionally influenced school functioning in youngsters that showed ED behaviors that were “out of character” (i.e., stubborn/irritable in youngsters with anxiety disorder). As learning to regulate emotions is primarily based on co-regulation with attachments figures, offering easy-access parent–child interaction interventions to reduce ED at preschool age may be considered an effective strategy to prevent a vicious cycle. Future research should focus on the environmental susceptibility of ED and interventions for high risk children and caregivers.

## Data availability statement

The original contributions presented in the study are included in the article/Supplementary material, further inquiries can be directed to the corresponding author.

## Ethics statement

The studies involving human participants were reviewed and approved by Karakter Child and Adolescent University Centre. Written informed consent to participate in this study was provided by the participants’ legal guardian/next of kin. In the methods section we describe how data was anonymized upon withdrawal from electronic health records.

## Author contributions

MB, NR, CH, and JB wrote the research proposal and data analysis plan. HK conveyed all data to SPSS. MB created the data set, executed data analysis, and wrote the manuscript. SD, NR, CH, and JB supervised the roles in this process. All authors contributed to the article and approved the submitted version.

## Funding

MB is part of the Eat2BeNice consortium (EU). Eat2BeNICE (H2020 grant agreement number 728018).

## Conflict of interest

The authors declare that the research was conducted in the absence of any commercial or financial relationships that could be construed as a potential conflict of interest.

## Publisher’s note

All claims expressed in this article are solely those of the authors and do not necessarily represent those of their affiliated organizations, or those of the publisher, the editors and the reviewers. Any product that may be evaluated in this article, or claim that may be made by its manufacturer, is not guaranteed or endorsed by the publisher.
