# Supplementary material for: Emotion dysregulation as cross-disorder trait in child psychiatry predicting quality of life and required treatment duration
Source: Front Psychiatry. 2023 Jul 20;14:1101226. doi: 10.3389/fpsyt.2023.1101226 (PMC10399689; doi:10.3389/fpsyt.2023.1101226)
Supplement: Supplementary file 1 [file Data_Sheet_1.docx]

**Supplementary information**

*Table S1. Child Behavior Checklist-EDI items*

| **No.** | **Description** | **Item** |
| --- | --- | --- |
| 1 | Argues a lot | 3 |
| 2 | Clings to adults or too dependent | 11 |
| 3 | Cries a lot | 14 |
| 4 | Deliberately harms self of attempts suicide | 18* |
| 5 | Destroys his/her own things | 20 |
| 6 | Destroys things belonging to his/her family or others | 21 |
| 7 | Gets in many fights | 37 |
| 8 | Nervous, high-strung, or tense | 45 |
| 9 | Too fearful or anxious | 50 |
| 10 | Physically attacks people | 57 |
| 11 | Screams a lot | 68 |
| 12 | Stubborn, sullen, or irritable | 86 |
| 13 | Sudden changes in mood or feelings | 87 |
| 14 | Talks about killing self | 91* |
| 15 | Temper tantrums or hot temper | 95 |
| 16 | Threatens people | 97* |
| 17 | Unhappy, sad, or depressed | 103 |
| 18 | Worries | 112 |

* Missing from Child Behavior Checklist 1.5-5 Preschool version

*Table S2. Preschool study sample flowchart*

*Table S3. School age study sample flowchart*

*Table S4. Sensitivity analysis of the full sample and subsample logistic regression coefficients in preschool and school age group*

| **CBCL preschool EDI total** | |  |  |  | **CBCL school age EDI total** | |  |  |
| --- | --- | --- | --- | --- | --- | --- | --- | --- |
| **ADHD** | | | |  | **ADHD** | | | |
| **full sample** | | **subsample** | |  | **full sample** | | **subsample** | |
| *Exp (B)* | *95% CI* | *Exp (B)* | *95% CI* |  | *Exp (B)* | *95% CI* | *Exp (B)* | *95% CI* |
| 1.0 | .98-1.0 | .97 | .95-.99 |  | .96 | .96-.97 | .91 | .90-.92 |
| **ASD** | | | |  | **ASD** | | | |
| **full sample** | | **subsample** | |  | **full sample** | | **subsample** | |
| *Exp (B)* | *95% CI* | *Exp (B)* | *95% CI* |  | *Exp (B)* | *95% CI* | *Exp (B)* | *95% CI* |
| .99 | .97-1.0 | .96 | .95-.98 |  | 1.0 | 1.0-1.0 | .99 | 1.0-1.0 |
| **ODD/CD** | | | |  | **ODD/CD** | | | |
| **full sample** | | **subsample** | |  | **full sample** | | **subsample** | |
| *Exp (B)* | *95% CI* | *Exp (B)* | *95% CI* |  | *Exp (B)* | *95% CI* | *Exp (B)* | *95% CI* |
| 1.1 | 1.1-1.1 | 1.1 | 1.0-1.1 |  | 1.0 | 1.1-1.1 | 1.0 | 1.0-1.0 |
| **Anxiety** | | | |  | **Anxiety** | | | |
| **full sample** | | **subsample** | |  | **full sample** | | **subsample** | |
| *Exp (B)* | *95% CI* | *Exp (B)* | *95% CI* |  | *Exp (B)* | *95% CI* | *Exp (B)* | *95% CI* |
| 1.1 | 1.0-1.1 | 1.0 | .98-1.0 |  | 1.0 | 1.0-1.0 | 1.0 | .98-1.0 |
|  |  |  |  |  | **Mood** | | | |
|  |  |  |  |  | **full sample** | | **subsample** | |
|  |  |  |  |  | *Exp (B)* | *95% CI* | *Exp (B)* | *95% CI* |
|  |  |  |  |  | 1.1 | 1.0-1.1 | 1.0 | .98-1.0 |

| *Table S5. Sensitivity analyses percentage CBCL-EDI is often/clearly present in the full sample and subsample for preschool and school age group* | | | | | | | | |  |  |  |
| --- | --- | --- | --- | --- | --- | --- | --- | --- | --- | --- | --- |
|  |  |  |  |  |  |  |  |  |  |  |  |
| **No.** | **Description** | **ADHD** | | **ASD** | | **ODD/CD** | | **Anxiety** | |  |  |
|  |  | **Full sample** | **Subsample** | **Full sample** | **Subsample** | **Full sample** | **Subsample** | **Full sample** | **Subsample** |  | |
|  |  | **N=612 (%)** | **N=396 (%)** | **N= 931 (%)** | **N=753 (%)** | **N=133 (%)** | **N=60 (%)** | **N=100 (%)** | **N=71 (%)** |  |  |
| 3 | Argues a lot | (5.9) | (5.8) | (9.8) | (10.3) | (9.8) | (11.6) | (5.0) | (2.6) |  |  |
| 11 | Clings to adults or too dependent | (19.1) | (17.0) | (25.9) | (25.5) | (27.8) | (40.6) | (41.0) | (42.1) |  |  |
| 14 | Cries a lot | (14.9) | (12.8) | (16.8) | (15.8) | (19.5) | (24.6) | (22.0) | (26.3) |  |  |
| 18 | Deliberately harms self of attempts suicide | - | - | - | - | - | - | - | - |  |  |
| 20 | Destroys his/her own things | (20.4) | (15.0) | (14.6) | (10.4) | (32.3) | (24.6) | (15.0) | (9.2) |  |  |
| 21 | Destroys things belonging to his/her family or others | (15.5) | (12.0) | (11.5) | (6.7) | (27.8) | (18.8) | (13.0) | (3.9) |  |  |
| 37 | Gets in many fights | (11.8) | (7.5) | (7.3) | (10.6) | (19.5) | (13.0) | (8.0) | (23.7) |  |  |
| 45 | Nervous, high-strung, or tense | (10.6) | (3.3) | (12.0) | (14.4) | (13.5) | (10.1) | (24.0) | (28.9) |  |  |
| 50 | Too fearful or anxious | (4.7) | (6.8) | (13.2) | (7.4) | (6.8) | (15.9) | (28.0) | (6.6) |  |  |
| 57 | Physically attacks people | (8.7) | (39.0) | (8.4) | (35.5) | (17.3) | (63.8) | (10.0) | (34.2) |  |  |
| 68 | Screams a lot | (42.8) | (47.0) | (38.1) | (45.5) | (63.2) | (73.9) | (37.0) | (48.7) |  |  |
| 86 | Stubborn, sullen, or irritable | (50.6) | (28.7) | (47.4) | (31.4) | (72.2) | (47.8) | (52.0) | (31.6) |  |  |
| 87 | Sudden changes in mood or feelings | (32.5) | (40.0) | (33.3) | (35.9) | (45.9) | (56.5) | (37.0) | (42.1) |  |  |
| 91 | Talks about killing self | - | - | - | - | - | - | - | - |  |  |
| 95 | Temper tantrums or hot temper | (42.2) | (5.3) | (37.5) | (7.0) | (60.2) | (11.6) | (45.0) | (6.6) |  |  |
| 97 | Threatens people | - | - | - | - | - | - | - | - |  |  |
| 103 | Unhappy, sad, or depressed | (3.1) | (17.0) | (3.9) | (25.5) | (6.8) | (40.6) | (7.0) | (42.1) |  |  |
| 112 | Worries | (5.9) | (12.8) | (7.3) | (15.8) | (9.0) | (24.6) | (9.0) | (26.3) |  |  |
| **No.** | **Description** | **ADHD** | | **ASD** | | **ODD/CD** | | **Anxiety** | | **Mood** | |
|  |  | **Full sample** | **Subsample** | **Full sample** | **Subsample** | **Full sample** | **Subsample** | **Full sample** | **Subsample** | **Full sample** | **Subsample** |
|  |  | **N=3821 (%)** | **N=1727 (%)** | **N=3590 (%)** | **N=1632 (%)** | **N=647 (%)** | **N=357 (%)** | **N=1056 (%)** | **N=238 (%)** | **N= 937 (%)** | **N=189 (%)** |
| 3 | Argues a lot | (33.5) | (27.4) | (34.1) | (31.6) | (58.3) | (54.8) | (24.7) | (20.5) | (27.2) | (23.4) |
| 11 | Clings to adults or too dependent | (23.2) | (18.5) | (30.4) | (28.9) | (24.3) | (18.1) | (33.4) | (32.8) | (21.5) | (12.6) |
| 14 | Cries a lot | (9.0) | (7.3) | (11.1) | (10.8) | (8.3) | (7.9) | (15.8) | (13.8) | (15.3) | (13.2) |
| 18 | Deliberately harms self of attempts suicide | (0.8) | (0.4) | (1.9) | (1.8) | (2.3) | (2.8) | (4.4) | (3.0) | (11.6) | (17.3) |
| 20 | Destroys his/her own things | (9.0) | (7.4) | (7.5) | (6.8) | (19.8) | (19.8) | (5.9) | (5.2) | (5.7) | (3.5) |
| 21 | Destroys things belonging to his/her family or others | (7.0) | (5.3) | (6.4) | (5.2) | (16.7) | (14.7) | (4.3) | (4.4) | (4.3) | (1.8) |
| 37 | Gets in many fights | (5.4) | (4.0) | (4.4) | (3.4) | (12.5) | (11.3) | (2.3) | (2.2) | (2.9) | (2.3) |
| 45 | Nervous, high-strung, or tense | (20.6) | (14.5) | (28.8) | (27.7) | (26.4) | (26.6) | (41.3) | (38.5) | (35.4) | (27.5) |
| 50 | Too fearful or anxious | (12.1) | (7.3) | (22.0) | (22.3) | (14.7) | (9.6) | (45.5) | (48.1) | (24.1) | (14.3) |
| 57 | Physically attacks people | (3.9) | (2.5) | (5.2) | (4.8) | (10.5) | (12.4) | (2.6) | (2.2) | (3.3) | (1.5) |
| 68 | Screams a lot | (28.9) | (24.4) | (26.9) | (24.2) | (45.9) | (42.9) | (19.6) | (15.8) | (18.2) | (14.9) |
| 86 | Stubborn, sullen, or irritable | (39.8) | (32.8) | (43.9) | (42.1) | (59.8) | (56.6) | (36.1) | (31.1) | (38.8) | (33.3) |
| 87 | Sudden changes in mood or feelings | (32.5) | (24.5) | (39.4) | (37.3) | (52.9) | (50.8) | (38.2) | (35.1) | (45.0) | (44.4) |
| 91 | Talks about killing self | (2.5) | (1.5) | (4.6) | (3.9) | (6.5) | (6.8) | (5.9) | (3.7) | (12.6) | (12.3) |
| 95 | Temper tantrums or hot temper | (30.8) | (24.8) | (33.8) | (33.0) | (54.3) | (57.1) | (25.3) | (23.5) | (22.9) | (20.5) |
| 97 | Threatens people | (2.0) | (1.2) | (2.4) | (2.1) | (7.6) | (7.3) | (1.8) | (1.0) | (1.6) | (1.8) |
| 103 | Unhappy, sad, or depressed | (10.9) | (6.0) | (19.0) | (16.3) | (21.6) | (22.0) | (32.9) | (28.6) | (53.3) | (55.3) |
| 112 | Worries | (14.4) | (10.2) | (23.7) | (22.7) | (17.6) | (19.8) | (41.3) | (40.5) | (48.2) | (46.8) |

*Figure S1. Kidscreen-27 subscale scores in norm groups and disorder categories*

*Figure S2. Relative predictive value of CBCL-EDI 1.5-5 total score and individual items across disorders in univariable regression analysis*

*
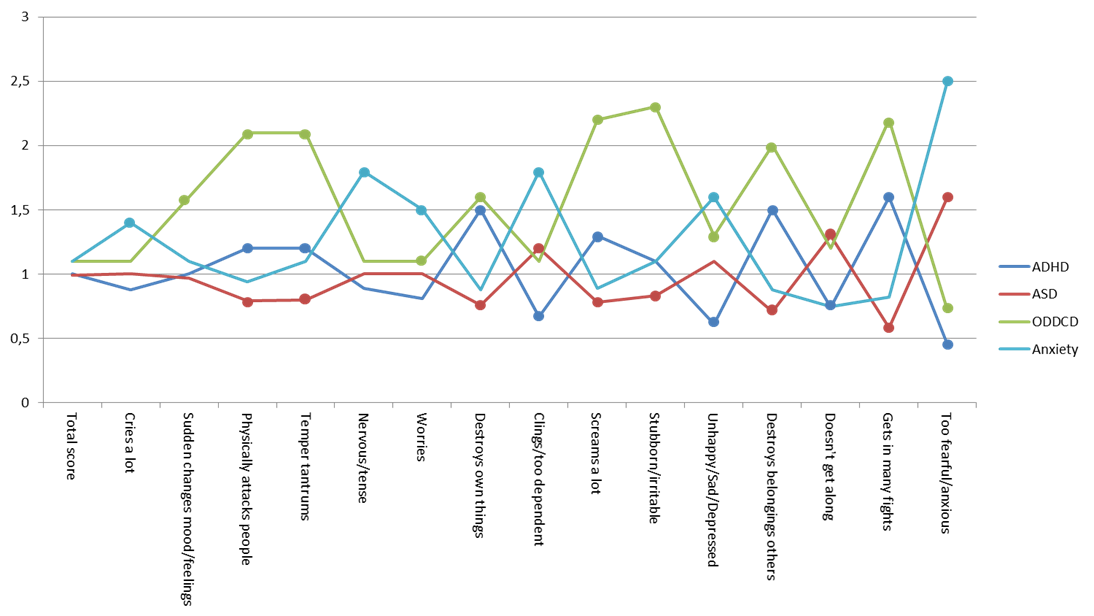
*

Numbers represent (B)exp in univariate regression analysis CBCL-EDI total score and individual items predicting disorder category (e.g. ADHD = 1, yes, ADHD = 0, no) versus all others

*Figure S3. Relative predictive value of CBCL-EDI 6-18 total score and individual items across disorders in univariable regression analysis*

*
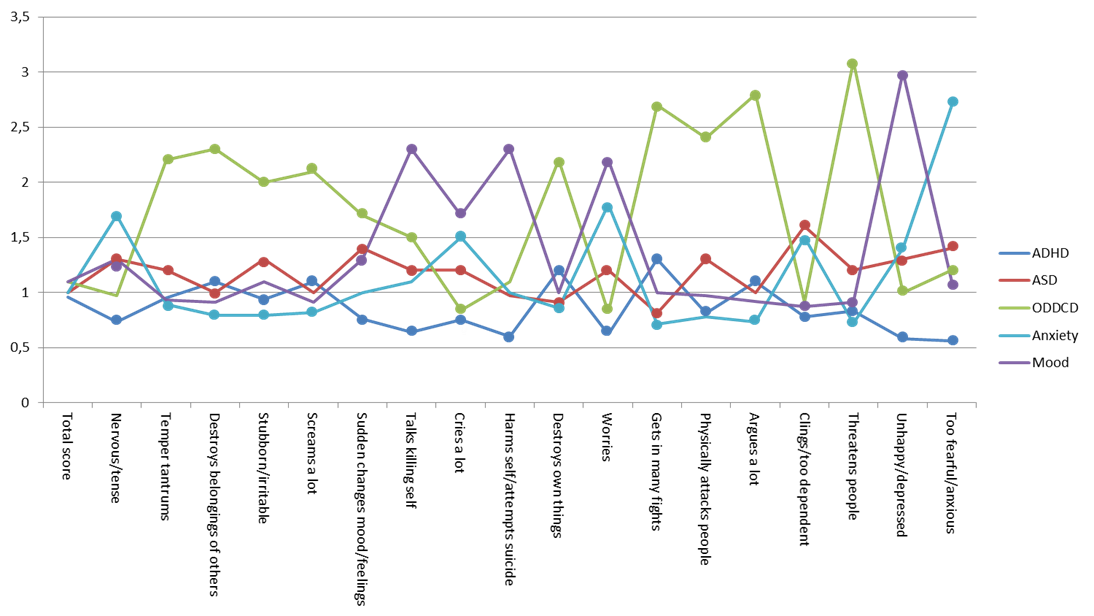
*

Numbers represent (B)exp in univariate regression analysis CBCL-EDI total score and individual items predicting disorder category (e.g. ADHD = 1, yes, ADHD = 0, no) versus all others
